# Monoclonal Antibody Therapy in Alzheimer’s Disease

**DOI:** 10.3390/pharmaceutics16010060

**Published:** 2023-12-29

**Authors:** Monica Neațu, Anca Covaliu, Iulia Ioniță, Ana Jugurt, Eugenia Irene Davidescu, Bogdan Ovidiu Popescu

**Affiliations:** 1 Department of Clinical Neurosciences, “Carol Davila” University of Medicine and Pharmacy, 050474 Bucharest, Romania; monica.neatu@rez.umfcd.ro (M.N.); anca.covaliu@rez.umfcd.ro (A.C.); iulia.ionita@rez.umfcd.ro (I.I.); ana.jugurt@rez.umfcd.ro (A.J.); bogdan.popescu@umfcd.ro (B.O.P.); 2 Department of Neurology, Colentina Clinical Hospital, 020125 Bucharest, Romania; 3 Department of Cell Biology, Neurosciences and Experimental Myology, “Victor Babeș” National Institute of Pathology, 050096 Bucharest, Romania

**Keywords:** Alzheimer’s disease, ß-amyloid, tau protein, passive immunotherapy, monoclonal antibodies

## Abstract

Alzheimer’s disease is a neurodegenerative condition marked by the progressive deterioration of cognitive abilities, memory impairment, and the accumulation of abnormal proteins, specifically beta-amyloid plaques and tau tangles, within the brain. Despite extensive research efforts, Alzheimer’s disease remains without a cure, presenting a significant global healthcare challenge. Recently, there has been an increased focus on antibody-based treatments as a potentially effective method for dealing with Alzheimer’s disease. This paper offers a comprehensive overview of the current status of research on antibody-based molecules as therapies for Alzheimer’s disease. We will briefly mention their mechanisms of action, therapeutic efficacy, and safety profiles while addressing the challenges and limitations encountered during their development. We also highlight some crucial considerations in antibody-based treatment development, including patient selection criteria, dosing regimens, or safety concerns. In conclusion, antibody-based therapies present a hopeful outlook for addressing Alzheimer’s disease. While challenges remain, the accumulating evidence suggests that these therapies may offer substantial promise in ameliorating or preventing the progression of this debilitating condition, thus potentially enhancing the quality of life for the millions of individuals and families affected by Alzheimer’s disease worldwide.

## 1. Introduction

### 1.1. Epidemiology of Alzheimer’s Disease

Dementia is an overarching syndrome primarily characterized by the gradual degradation of cognitive abilities across various domains, leading to compromised daily functioning across social, physical, and occupational dimensions [1,2,3]. Presently, approximately 35.6 million individuals worldwide are afflicted with dementia, with a yearly incidence of 7.7 million novel cases [4,5]. Recent empirical investigations corroborate this trajectory and foresee an anticipated 87% increase in Europe spanning the years 2010 to 2050 due to the influence of the “baby boomer” phenomenon [4,6].

Alzheimer’s Disease (AD) is a neurodegenerative disorder that stands as the most predominant form of dementia, including 60–80% of reported cases, thereby imposing a marked burden on both domestic and international healthcare systems [7,8]. In the contemporary era, various health conditions, including cardiovascular disease, have demonstrated a decrease, unlike AD, which has experienced a surge of 68% in the last decade [9,10]. Research examining AD indicates that among individuals aged 45–64 years, the annual prevalence is approximately 24.2 per 100,000, with an incidence rate of 6.3 per 100,000 [11]. Nevertheless, the prevalence of the disease is notably higher in individuals who are aged over 65 years, and the probability of AD development experiences an exponential growth with advancing age, doubling approximately every five years thereafter, impacting 3% of individuals aged 65–75 and 32% of those aged above 84 years [6,11,12,13,14,15].

### 1.2. Physiopathology of AD

Unfortunately, a conclusive diagnosis of Alzheimer’s disease is only achievable through histopathological examination of the brain tissue. To diagnose this condition, the presence of distinct characteristics is required: amyloid pathology, tau pathology and neuroinflammation, neuronal death, as well as brain atrophy [16,17]. 

#### 1.2.1. Amyloid-β Pathology

Amyloid β (Aβ) precursor protein (APP) is one of the polypeptides predominantly present in the central nervous system (CNS) [18]. Aβ originates from the enzymatic cleavage of the APP protein [16]. This cleavage, facilitated by β- and µ-secretases, gives rise to different products, one of which is the Aβ [16,19,20]. Among the potential variations of Aβ, Aβ40 and Aβ42 stand out as the prevalent ones, with Aβ42 being the most prone to form fibrils and exhibit toxicity. Aβ monomers tend to aggregate, resulting in the creation of dimers, trimers, and larger assemblies. These eventually lead to the development of senile plaques (SP), which are deposited in the extraneuronal regions [21]. SP typically emerge first in the parietal cortex and subsequently extend to other regions [22]. These plaques are a distinctive feature of AD and are closely associated with cognitive decline and neuronal damage [23,24]. At the synaptic level, Aβ interferes with neurotransmission [25]. The individual units within Aβ hinder neurotransmitter receptors and disrupt the proper functioning of the sodium–potassium pump, impeding both electrical and chemical transmission [16,25]. This disruption obstructs the accurate interneuronal connections [25]. Furthermore, this peptide encourages the internalization of channels linked to neurotransmitter receptors. When this circumstance persists, it leads to synaptic dysfunction and the subsequent deterioration of neuronal synapses. Compact SP may also alter cortical synaptic integration [26,27,28,29,30]. 

It is worth mentioning that the peptide Aβ40 is notably inclined to build up within the arterial structures of the cerebrovascular and leptomeningeal systems, a phenomenon known as Cerebral Amyloid Angiopathy (CAA) [31,32,33]. This alteration is present in as many as 90% of AD patients but can also manifest in individuals without AD [16,34,35]. The consequence of CAA is a reduction in blood flow through the impacted vessels, leading to an elevated occurrence of cerebral ischemia [36]. Furthermore, the removal of surplus Aβ monomers can occur by excreting these peptides into the bloodstream [37]. In the context of AD, the proper elimination of vascular Aβ becomes impeded, thus encouraging the buildup of deposits, resulting in the formation of CAA within the vessels [16].

A different pathway for the degradation of Aβ monomers involves neuronal enzymes [38]. Neprilysin, a transmembrane peptide, possesses the ability to break down Aβ dimers [19]. Despite being synthesized by neurons, no heightened production of neprilysin has been observed in Aβ pathology [38]. In this regard, certain studies suggest that augmenting the synthesis of this enzyme could enhance neuronal safeguarding and survival in the context of AD [38]. Moreover, it is known that the insulin-degrading enzyme can also break down Aβ monomers [39,40]. This protein, synthesized by neurons, displays altered production in AD patients [41,42]. Its potential role as a bridge connecting AD and diabetes mellitus has been proposed, attributable to its dual capacity to degrade both Aβ and insulin [16,43]. 

In Alzheimer’s disease, the initial phase involves the buildup of Aβ in the medial parietal cortex [22]. However, in individuals without AD, the concentration of neurofibrillary tangles (NFTs) in the medial temporal lobe precedes the accumulation of Aβ [16,22]. It is worth noting that the existence of Aβ deposits does not exclusively correlate with neurodegenerative processes [44]. In contrast, tau pathology plays a more significant role in driving neuronal demise and brain atrophy, as elaborated below [44].

#### 1.2.2. Tau Pathology

The tau protein is a small molecule that binds to microtubules, providing them essential stability [16,18]. These microtubules play a critical role in maintaining the neuron configuration, facilitating axon transport, and contributing to synaptic plasticity [18]. When the tau protein undergoes improper phosphorylation, the structure of microtubules undergoes alteration [22]. Phosphorylated tau proteins initiate the phosphorylation process in additional tau proteins, propagating within a neuron and spreading to adjacent neurons via neuronal synapses [22]. Neurofibrillary tangles predominantly consist of hyperphosphorylated tau protein and form filamentous aggregates within neuronal cell bodies and proximal dendrites [45,46]. Therefore, the hyperphosphorylation of tau protein alters the neural junctions, causing cellular changes that result in the loss of synapses, the branching of neurons, and ultimately neuronal loss [18]. It is established that the buildup of NFTs triggers cell death, leading to the formation of aggregates of the hyperphosphorylated protein known as neuronal ghosts [47]. Unlike senile plaques, which are distributed proportionally across various affected regions in AD, neurofibrillary tangles are primarily concentrated in the entorhinal cortex and hippocampus; the posteromedial subregion of the entorhinal cortex is responsible for spatial memory, while the anterolateral area is linked to episodic recollection [16,22,47]. Consequently, it is reasonable to conclude that the progression of intraneuronal NFT deposition closely parallels neuronal loss and subsequent brain atrophy and cognitive decline [16].

#### 1.2.3. Neuroinflammation and Neuronal Death

Neuroinflammation also presents a significant contribution to the progression of neurodegenerative processes [48,49]. Microglia, functioning as macrophages within the brain, represents the initial defense system of the CNS [50,51]. They possess phagocytic capabilities that enable them to clear harmful substances from the environment and trigger tissue inflammation [50,51,52]. Inflammation is originally a defensive reaction, yet its prolonged state can be harmful to the tissue [52,53,54]. Consequently, brain inflammation becomes a distinctive feature of Alzheimer’s disease [54]. As early events in AD’s pathophysiology unfold, including the increase in Aβ levels even before the formation of senile plaques, microglial activation has been observed [55]. Subsequently, when senile plaques do manifest, microglial cells migrate towards these plaques and enclose them within the first 24 h [16,56]. While they succeed to surround and contain their expansion, these microglia do not seem to effectively phagocytize the plaques, which endure over the course of the individual’s life [56,57].

This phenomenon maintains microglia’s heightened activity from the onset of AD, shaping a pattern of proinflammatory cytokines that fosters a milieu of neurotoxic oxidative stress [58,59]. Furthermore, an enduring proinflammatory state diminishes the capacity to clear Aβ, consequently exacerbating the progression of AD [60]. This comprehensive pathogenesis culminating in neuronal death adheres to a sequential timeline [23]. The initial notable occurrence often involves the excessive production of various isoforms of Aβ, with associated toxicity, including those driven by the harmful impacts of neuroinflammation [16,23]. The subsequent buildup of NFTs typically follows, serving as the second event preceding neuronal death, which ultimately leads to cerebral atrophy in individuals affected by AD [16,23].

### 1.3. Clinical Forms and Progression of AD 

In Alzheimer’s disease, the initial harm occurs in the cerebral areas that play a pivotal role in memory, language, and cognitive functions. Consequently, the primary manifestations typically involve difficulties with these cognitive abilities. While these symptoms may be new to the affected person, the underlying brain alterations believed to be responsible for them are assumed to commence two decades or more prior to the onset of noticeable symptoms [61,62]. As the disease advances, the spread of neuropathology persists, leading to a substantial reduction in total brain mass, which can reach up to 35% [63,64].

**Figure 1:**
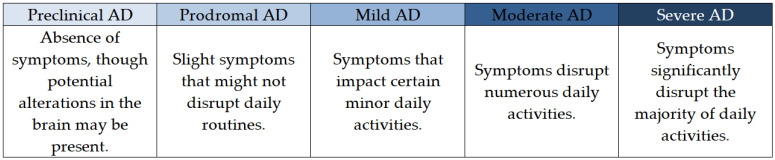
AD stages [61].

Within this AD continuum, three overarching phases exist: preclinical Alzheimer’s disease, mild cognitive impairment (MCI) caused by Alzheimer’s disease, and Alzheimer’s dementia, which includes stages of mild, moderate, and severe dementia (Figure 1) [61,62,65].

Although the commencement of the Alzheimer’s disease continuum at preclinical Alzheimer’s disease (absence of symptoms) and its culmination at severe Alzheimer’s dementia (intense symptoms) are understood, the duration patients spend in each segment of this spectrum fluctuates. The duration of every segment is contingent on factors such as age, genetic predisposition, biological sex, and various other influences [62,63,64,65,66,67].

## 2. Results

Following a concise overview of Alzheimer’s disease pathology, we will explore the primary therapeutic antibodies employed in its treatment. Furthermore, we will categorize these molecules based on their mode of action: antibodies directed towards the amyloid pathway components and those focused on the tau pathway.

### 2.1. Amyloid-β Pathology

#### 2.1.1. Aducanumab

**Figure 2:**
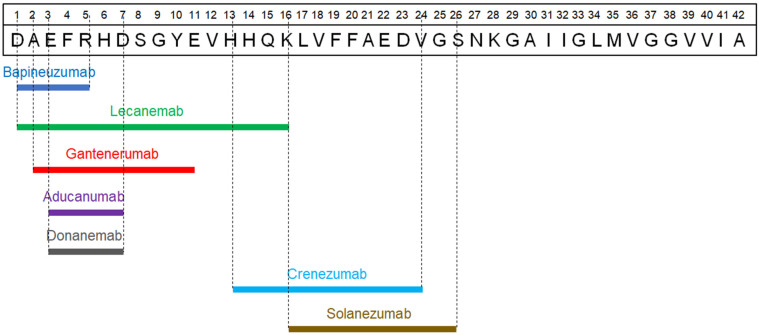
Comparative analysis of specific antibody epitopes [67].

Aducanumab (BIIB037) is a human antibody of the IgG1 class designed to attach to the N terminus of Aβ in an elongated conformation. As per both structural and biochemical examinations, it has been established that aducanumab forms a binding interaction with the linear epitope created by Aβ amino acids 3–7. The primary contact between Aβ and the Fab region of aducanumab is facilitated by specific Aβ residues, namely, Glu3, Phe4, Arg5, His6, and Asp7 [68]. Its purpose is to specifically target Aβ aggregates, encompassing both soluble oligomers and insoluble fibrils [64,66,67] (Table 1) (Figure 2).

Aβ disposal involves microglia that binds to the Fc part of the antibody, which enhances the phagocytosis of aducanumab–Aβ complexes [69,70]. Additionally, aducanumab exerts a neuroprotective effect by limiting the toxicity of Aβ oligomers. This is achieved by obstructing the attachment of soluble Aβ oligomers to metabotropic receptors and by delaying their discharge into the neuropil from plaques, thus averting neurotoxicity induced by calcium [69,71]. In the phase Ib randomized trial called PRIME (NCT01677572), significant reductions in the composite score of the amyloid positron emission tomography (PET) standard uptake value ratio (SUVRr) were observed in patients who received aducanumab, especially those who were administered a dose of 10 mg/kg over a 54-week duration [67,70]. Among individuals with prodromal or mild AD, the decrease in the brain amyloid burden exhibited a pattern that was both dose-dependent and influenced by the duration of treatment. Additionally, the administration of aducanumab resulted in a delay in the Clinical Dementia Rating-Sum of Boxes (CDR-SB) and Mini Mental State Examination (MMSE) scores, indicating a favorable impact on cognition and the clinical progression of the condition [67,70]. Aducanumab delivered compelling outcomes in the PRIME trial, although the incidence of amyloid-related imaging abnormalities vasogenic edema (ARIA-E) exhibited a dose-dependent pattern, affecting 3% to 41% of individuals receiving aducanumab. Notably, ARIA-E was more prevalent among those carrying the APOE ε4 gene variant [67,70].

In the EMERGE study (NCT02484547), a phase III randomized clinical trial, there was a notable 22% reduction in CDR-SB scores, along with significant enhancements in various assessments including the MMSE, the Alzheimer’s Disease Assessment Scale-Cognitive Subscale (13 Items) (ADAS-Cog 13), and the Alzheimer’s Disease Cooperative Study-Activities of Daily Living Inventory (Mild Cognitive Impairment Version) (ADCS-ADL-MCI) following the administration of aducanumab at a monthly dose of 10 mg/kg IV [72,73]. The ENGAGE study, another phase III randomized clinical trial, failed to replicate these findings, likely because of distinctions in study parameters, the progression of the disease, and varying responses depending on the quantity of treatments administered [72,74]. In both trials, significant reductions in amyloid deposition were observed in subgroup analyses for participants who received high-dose aducanumab treatment (10 mg/kg) compared to those who received a placebo. However, noteworthy reductions in cerebrospinal fluid (CSF) tau proteins were only documented in the EMERGE study [72,73,74]. When looking at the combined data from both the EMERGE and ENGAGE trials, it becomes evident that ARIA (Amyloid-Related Imaging Abnormalities), including both edema and hemorrhage (ARIA-H), was quite prevalent, occurring in 41.3% of individuals who received aducanumab at 10 mg/kg. However, the occurrence of symptomatic ARIA was relatively low, with only around 20% of radiographically identified ARIA cases manifesting mild adverse reactions, usually in the form of headaches [69,75,76]. Severe adverse ARIA incidents such as delirium, deterioration in memory, and seizures were infrequent, with similar occurrence rates regardless of the individual’s APOE ε4 genotype, at 1.5% in the EMERGE trial and 1.4% in the ENGAGE trial [75,76].

The pharmacokinetics (PK) of aducanumab in early Alzheimer’s disease patients were effectively described. The model accurately represented individual concentration-time profiles for relevant dose-titration regimens, demonstrating robustness through model diagnostics and visual predictive checks (VPCs). The estimated half-life of aducanumab was approximately 24.8 days, aligning with similar antibodies in the IgG1 subclass.

Body weight was identified as a significant factor affecting the clearance, the volume of distribution in the central compartment, and the volume of distribution in the peripheral compartment. A 10% increase in body weight correlated with a 3–5% increase in the clearance, central, and peripheral compartment. The final PK model exhibited low residual error values, indicating high precision and minimal unexplained variability, mostly attributed to assay measurement errors.

Utilizing the PK model, the study compared the number of patients reaching the steady state with at least 10 uninterrupted infusions. Around 30% of patients across both studies did not achieve the steady state, with the study EMERGE showing a higher percentage than the study ENGAGE. Although post hoc PK parameters did not reveal differences between the studies ENGAGE and EMERGE, alterations in dose and dosing interruptions affected the duration patients spent in the steady state, potentially impacting pharmacology. Moreover, SUVR was assumed constant over time for placebo patients, considering minimal changes in amyloid levels in early Alzheimer’s patients.

Age was identified as a significant factor influencing the change in SUVR over time, with older patients showing a larger effect, possibly due to a leaky blood–brain barrier. The exposure-Aβ removal relationship and SUVR-time profiles for different doses highlighted the importance of both the magnitude and duration of exposure in Aβ removal [77].

The US Food and Drug Administration (FDA) granted approval for this medication in April 2021 because it is believed to have the capacity to target senile plaques, thereby slowing down the advancement of Alzheimer’s disease. The approval rested upon findings from three clinical trials, collectively presenting robust evidence supporting the efficacy of this therapy [72]. It is important to note that aducanumab has become the first newly approved drug for the prodromal and mild form of AD by the FDA since the launch of memantine in 2003 [67,72] (Table 2).

#### 2.1.2. Lecanemab

Lecanemab (BAN2401) is an IgG1 humanized antibody that exhibits a strong affinity for binding to soluble Aβ aggregates, including oligomers and protofibrils, displaying significant selectivity when compared to both monomers and insoluble fibrils. It exerts its pathophysiological action by attaching to the N-terminus segment of the Aβ protein, specifically binding to the amino acids 1–16 [69] (Table 1).

During its preclinical development, lecanemab demonstrated efficacy in diminishing Aβ protofibrils in both the brain and cerebrospinal fluid (CSF) of Tg-ArcSwe mice. The incorporation of the Arctic mutation alongside the Swedish mutation in the transgene led to an intensified Aβ pathology, characterized by heightened levels of soluble Aβ aggregates, particularly Aβ protofibrils. This combination also resulted in a more substantial accumulation of Aβ within neurons and the formation of more robust senile plaques compared to scenarios involving the Swedish mutation alone. Follow-up investigations conducted in mouse neuron-glial co-cultures indicated that lecanemab could potentially safeguard neurons by mitigating the toxicity associated with Aβ protofibrils. This protective effect seemed to be achieved by antagonizing the pathological buildup of these protofibrils in astrocytes [79].

A phase 1 clinical trial, identified as NCT01230853 and conducted from 2010 to 2013, was designed to assess the safety and tolerability of lecanemab. This trial followed a multicenter, double-blind, randomized, and placebo-controlled format [69]. It specifically enrolled patients who had mild-to-moderate dementia attributed to Alzheimer’s disease, with an average MMSE score of 23 [80,81]. Lecanemab was administered in both single and multiple ascending doses during the study, ranging from 0.1 mg/kg as a one-time dose to 10 mg/kg administered twice a week for a duration of 4 months [69,81,82]. Notably, the occurrence of ARIA was relatively low in this study [81,82]. Asymptomatic ARIA-H was noted in 5% of participants treated with lecanemab and in 10% of those who received a placebo [80,81].

Lecanemab underwent further evaluation in a distinctive Phase 2b clinical trial (NCT01767311) conducted from 2012 to 2017. This study spanned a duration of 18 months and adopted a multicenter, double-blind, and placebo-controlled design. It specifically enrolled individuals with Mild Cognitive Impairment (MCI) and mild dementia attributed to Alzheimer’s disease [69,82]. Participants were confirmed to have Alzheimer’s disease based on a positive brain Aβ PET scan. The study incorporated a total of 854 participants, with a mean MMSE score of 25.6. The study incorporated an innovative design aimed at enhancing efficiency that allowed for the dynamic allocation of participants across six different treatment arms. These arms included a placebo group and five different lecanemab dosing regimens: 2.5 mg/kg administered twice a week, 5 mg/kg administered monthly, 5 mg/kg administered biweekly, 10 mg/kg administered monthly, and 10 mg/kg administered twice a week. This approach aimed to optimize the allocation of participants based on evolving response data [69]. The main goal of the study was to ascertain the ED90 target dose. This target dose refers to the specific treatment dosage that would produce a therapeutic effect equal to or greater than 90% of the maximum expected treatment effect, as predicted by the model at the 12-month mark. The study aimed to achieve this objective with a probability of at least 80%, signifying a high level of confidence in identifying the most effective dose. Individuals were randomly assigned in a 3:1 ratio to receive one of the five lecanemab treatment regimens or a placebo. Upon assignment to a particular regimen, participants maintained their affiliation with that group throughout the entire study period [69,80,81]. Nonetheless, the distribution of subsequent participants into distinct treatment groups was adapted in response to regular interim analyses. These analyses aimed to determine which treatment regimen had the highest likelihood of being the most effective dosage—specifically, one that outperformed the placebo in reducing the primary clinical endpoint by at least 25%. In the study, the primary clinical endpoint under examination was the change in the AD composite score (ADCOMS) measured at the 18-month mark [69,80,81]. ADCOMS is a comprehensive clinical score that assesses cognitive and functional abilities by incorporating elements from various assessments, including ADAS-Cog, MMSE, and CDR [69,80,81].

During the course of this trial, regulatory guidance led to a decision that individuals carrying the APOE ε4 gene variant (70% of the individuals included in the trial) could not be enrolled in the highest lecanemab dose group. This precaution was taken due to concerns about an increased risk of ARIA. These actions resulted in a substantial disparity in the count of APOE ε4 carriers in the 10 mg/kg biweekly group, potentially impacting the study’s outcomes [69,80,81].

However, there were notable trends indicating a potential dose-response relationship in terms of clinical efficacy. Lecanemab demonstrated a dose-dependent reduction in brain Aβ, with the most significant decrease observed in the group receiving lecanemab at 10 mg/kg biweekly [69,80,81]. This even led to several participants transitioning to a status of brain amyloid PET negativity based on visual assessment [81] (Table 3).

Moreover, the 18-month treatment with lecanemab at 10 mg/kg biweekly resulted in a reduction in clinical decline when compared to the placebo group, as evidenced by improvements in ADCOMS (27% reduction), CDRsb (33% reduction), and ADASCog14 (56% reduction) [81] (Table 4).

It is important to note that the incidence of ARIA-E with lecanemab at 10 mg/kg biweekly was 9.9%, whereas with the placebo, it was 0.8%. Additionally, among participants receiving lecanemab, both ARIA-E and ARIA-H were more common in APOE ε4 carriers compared to noncarriers [69,81,82].

It is important to note that lecanemab displayed distinctive pharmacokinetic (PK) characteristics. Unlike numerous monoclonal antibodies that typically exhibit a nonlinear process due to target-mediated drug disposition (TMDD), lecanemab did not demonstrate TMDD, likely due to the predominant localization of its antigens (Aβ oligomers, protofibrils, and plaques) in the brain. Comparisons with other monoclonal antibodies used in AD revealed that lecanemab’s half-life for the typical patient is around 9.5 days, resembling that of donanemab but being shorter than that of aducanumab and gantenerumab [84,85] (Table 5).

Lecanemab’s clearance and volume increased with a higher body weight, and both parameters were slightly lower in women compared to men. The reason for these gender-based differences is unclear, though variations in the lymph flow rate and Fc receptor expression may contribute. Additionally, lecanemab’s clearance declined with increasing albumin levels [84,85].

Age and APOE4 carrier status, recognized risk factors for AD, did not have a significant impact on lecanemab PKs [84,85].

Lecanemab treatment, leading to the removal of amyloid plaques in the brain, was associated with an increase in Aβ42 and a decrease in p-tau181 in CSF. Changes in the plasma Aβ42/40 ratio and p-tau181 mirrored alterations in CSF Aβ42 and p-tau181. Amyloid PET SUVr and plasma biomarkers exhibited dose- and time-dependent changes. During the open-label extension gap period, with subjects off treatment for an average of 2 years, amyloid plaque levels re-accumulated slowly [84,85].

The dose-dependency in the decrease in SUVr and p-tau181 and the increase in the Aβ42/40 ratio were illustrated for 10 mg/kg biweekly and 10 mg/kg monthly after 18 months of treatment. Subjects receiving 10 mg/kg biweekly showed a faster and larger decrease in both SUVr and p-tau181, with a higher percentage achieving amyloid negativity for SUVr compared to subjects receiving 10 mg/kg monthly. Simulations suggested that continuous treatment at 10 mg/kg biweekly resulted in further SUVr decline, while discontinuation after 18 months led to slow re-accumulation over 15 years [84,85].

Based on these compelling findings, lecanemab received FDA approval on 6 January 2023 for the treatment of MCI or mild AD dementia [69,86,87]. Much like aducanumab, lecanemab obtained accelerated approval via the expedited clearance pathway. This pathway is reserved for situations with a critical medical demand, and the drug exhibits an influence on a surrogate endpoint that reliably anticipates clinical benefits [69,80,81,82,83,84,85,86,87].

Furthermore, there are other studies involving lecanemab, including a 5-year Phase 2 long-term extension and a 4-year Phase 3 long-term extension study focused on early AD [69,86]. Additionally, there is a 4-year trial within the Dominantly Inherited Alzheimer Network Trials Unit (DIAN-TU), referred to as the next-generation trial, for individuals with autosomal-dominant genetic AD, which will be discussed later [80,81,82,83,84,85,86,87].

#### 2.1.3. Donanemab

Donanemab (LY3002813) is a humanized monoclonal IgG1 antibody designed to selectively target the N-terminal pyroglutamate Aβ p3–7 epitope, a feature found exclusively in deposited Aβ [67,88].

In patients with early symptomatic Alzheimer’s disease, a phase II trial (TRAILBLAZER-ALZ) showed that the administration of donanemab led to a slight reduction in cognitive and functional decline compared to the placebo [89]. Furthermore, the findings from 18F-florbetapir PET scans indicated that individuals who received donanemab treatment experienced a notable decrease in amyloid plaque levels by week 76, with 54.7% of the people attaining a status free of amyloid by week 52 [67,89]. Moreover, during the first 12 weeks of donanemab administration, there was a swift decrease in plasma P-tau217, which is a biomarker indicative of Alzheimer’s disease pathology [67]. This treatment was linked to the occurrence of ARIA, which was observed in 40% of the participants who received donanemab. Among those experiencing ARIA, approximately 26.1% exhibited symptoms. On the other hand, symptomatic ARIA-E was observed in only 0.8% of participants who received the placebo [69,89,90].

In the TRAILBLAZER-ALZ 2 study, a phase III trial evaluating donanemab in MCI and mild dementia due to AD revealed promising results: a 35% reduction in the rate of cognitive and functional decline compared to the placebo, as measured by the primary endpoint (integrated Alzheimer’s disease rating scale—iADRS). The secondary endpoint (CDRsb) also showed a deceleration in decline by 36%, observed over an 18-month period, with donanemab treatment. Participants treated with donanemab experienced a 39% reduced likelihood of advancing to the subsequent disease stage, as indicated by the CDR global score, in comparison to those on placebo. Donanemab exhibited a 40% decrease in the deterioration of daily living abilities over the course of 18 months. Notably, a significant proportion of participants with intermediate tau levels achieved amyloid elimination, with 34% at 6 months and 71% at 12 months, based on the amyloid PET study. The incidence of symptomatic ARIA-E was 6.1% in the donanemab treatment group, while ARIA-H occurred in 31.4% of the participants receiving donanemab [69].

Other phase III trials (such as TRAILBLAZER-ALZ 3 and 4) showed that the safety profiles of aducanumab and donanemab treatments remained consistent with their previously published studies. In the case of donanemab, the incidence of ARIA was 25.4%, with 2.8% of cases being symptomatic (all ARIA-E). Meanwhile, aducanumab exhibited an ARIA incidence of 26.1%, with 4.3% of cases being symptomatic (all ARIA-E). Significantly, the occurrence of ARIA is notably reduced compared to the observations in TRAILBLAZER-ALZ. This variance might be ascribed to differences in the timing of adverse event evaluations: 76 weeks in TRAILBLAZER-ALZ and 24 weeks in TRAILBLAZER-ALZ 4 [69,91]. 

When it comes to the pharmacokinetics of donanemab in individuals with early symptomatic Alzheimer’s disease, its estimated half-life for the typical participant was 11.8 days, falling within the lower range of human IgG1 (18–21 days) and monoclonal antibodies. Minimal accumulation occurred with dosing every 4 weeks, achieving steady-state exposures after a single dose [92].

Body weight was identified as a significant covariate affecting total body and distributional clearances, as well as central and peripheral volumes of distribution. Clearance and volume followed typical allometric scaling, suggesting that heavier participants would experience a higher clearance and volume, resulting in a lower overall exposure. However, this weight-based impact on exposure was expected to be minimal, as serum concentrations remained above the threshold for amyloid clearance, even in participants with a higher body weight [92].

It was indicated that the majority of participants maintained serum concentrations above the threshold required for plaque removal, even in the presence of varying anti-drug antibody titers (ADA). The ADA titer had a minimal impact on the percentage of participants achieving plaque clearance [92].

The exploration of ARIA-E revealed an increased likelihood of ARIA-E events with donanemab treatment, primarily driven by participants carrying the APOE ε4 gene. This association highlights the importance of considering APOE ε4 status when administering amyloid-targeting therapies. The ARIA-E analysis was based on data from a single dosing regimen, limiting the exposure range for informative exposure–ARIA relationships [92,93].

#### 2.1.4. Crenezumab

Crenezumab (MABT5102A or RG7412) is a humanized IgG4 monoclonal antibody characterized by its strong binding affinity to both oligomeric and fibrillar forms of Aβ. This binding propriety of this molecule promotes the uptake of Aβ by microglia [94,95]. The antibody was purposefully designed to lower the chances of Fcγ receptor-triggered hyperactivity in microglia, a situation that could potentially result in neuroinflammation, vasogenic edema, and cerebral microhemorrhage. Crenezumab’s epitope is located on the central segment of the Aβ peptide (amino acids 13–24), and it adheres to aggregated forms while exhibiting disassembling properties [93,94,95,96]. In hAPP (human Amyloid Precursor Protein)/PSN1 (presenilin 1) transgenic mice, this immunotherapy approach effectively facilitated the removal of APCs (antigen-presenting cells), resulting in the diminished activation of the stress-associated p38 mitogen-activated protein kinases (P38 MAPK) in microglia and a minimal secretion of the proinflammatory cytokine TNF α (Tumor Necrosis Factor Alpha) [93,94,95,96].

The Phase I trial for crenezumab assessed the safety and the extent to which it could be tolerated when administered as a single intravenous dose (ranging from 0.3 to 10 mg/kg) or as multiple weekly doses with increasing amounts (ranging from 0.5 to 5 mg/kg). Notably, no negative effects/reactions to vasogenic edema, microhemorrhage, or ARIA were detected during the study [93,96,97].

The Phase II clinical study assessed the safety and effectiveness of crenezumab in two different dosing regimens: subcutaneous administration at a dose of 300 mg every two weeks and high-dose intravenous administration at 15 mg/kg every four weeks. The trial extended for a duration of up to 68 weeks and involved 431 patients diagnosed with mild-to-moderate Alzheimer’s disease, aged between 50 and 80 years [93,96,97]. The patients exhibited a notable rise in CSF Aβ42 levels, which had a connection with the levels of crenezumab in the CSF [13,98]. The concept here is that as crenezumab binds to Aβ, forming complexes, these complexes have a longer half-life than free Aβ. This phenomenon results in a sustained reduction in free Aβ levels, even at higher doses of crenezumab, leading to the observed non-dose-proportional increase in total Aβ levels. However, it is worth noting that the primary and secondary outcome measures related to cognitive decline were not achieved, and there were no observable significant trends suggesting a decelerated accumulation of Aβ [13,94,97]. The safety evaluation of crenezumab was found to be satisfactory, as only a single instance of ARIA suggestive of vasogenic edema or effusions was observed [94].

A post hoc analysis revealed that the high-dose group experienced a reduction in cognitive decline quantified by the ADAS-Cog scale [67]. Nevertheless, it is crucial to emphasize that high-dose Phase III trials of crenezumab (NCT02670083-CREAD and NCT03114657-CREAD2), involving intravenous infusion at a dosage of 30 mg/kg every 4 weeks for a duration of 100 weeks, in patients diagnosed with prodromal-to-mild sporadic Alzheimer’s disease, were terminated in January 2019 [67]. 

A separate Phase II study (NCT01998841) is presently assessing crenezumab in individuals carrying the familial early-onset mutation PSEN1 E280A [94]. In this context, participants are administered crenezumab either every two weeks or four weeks subcutaneously or intravenously for a minimum duration of 260 weeks [67,94]. 

In a recent Phase Ib study (NCT02353598), the safety, tolerability, and pharmacokinetics of crenezumab were assessed in 71 patients aged 50 to 90 years with mild-to-moderate Alzheimer’s disease. The study involved the administration of crenezumab at various doses (30, 45, 60, or 120 mg/kg) via intravenous infusion every four weeks for a duration of 13 weeks [94]. Approximately 94% of the participants experienced at least one mild or moderate adverse incident during the study, and 15.5% of them reported severe adverse events. Notably, there were no instances of ARIA, edema, or effusion observed. However, one case of ARIA, microhemorrhages, and hemosiderosis was noted in the 60 mg/kg dose group, and the study reported three severe adverse reactions, including malignant melanoma, unintentional overdose, and pneumonia [94,99].

In this combined analysis of data, the PK of crenezumab in patients with mild-to-moderate Alzheimer’s disease was found to be dose-proportional within the tested dose range. The clearance and half-life values of crenezumab were consistent with those expected for IgG monoclonal antibodies [100].

Body weight influenced elimination clearance, the intrinsic clearance of the crenezumab-Aβ complex, and the central distribution volume, increasing with a higher body weight [100].

Each administration of crenezumab led to a significant increase in total plasma Aβ40 and Aβ42 levels, indicating engagement with monomeric Aβ. The increase in total Aβ levels was dose-dependent but not dose-proportional, reaching a plateau at the 120 mg/kg dose. It was successfully explained that higher doses maintain reductions in free Aβ levels [100].

The analysis indicated that one’s age and glomerular filtration rate (GFR) are factors contributing to between-subject variability in baseline Aβ levels. Patient factors like age or sex did not significantly affect the dissociation constant, implying the independence of these factors in crenezumab-Aβ binding [100].

The PK data confirmed peripheral target engagement by crenezumab at evaluated doses, complementing the CNS target engagement observed in the previous studies. The simulation suggested that circulating crenezumab is mostly unbound, with the transfer to peripheral organs and the CNS unaffected by Aβ binding. Predicted free Aβ levels continued to decrease with increasing doses, even after the total Aβ reached a plateau, indicating the importance of considering free Aβ kinetics in assessing drug effects [100].

#### 2.1.5. Bapineuzumab

Bapineuzumab (AAB-001) is a humanized murine monoclonal IgG1 antibody derived from the murine IgG2a antibody 3D6 targeting the N-terminal region of β-amyloid (residues 1–5) [101,102]. This anti-3D6 antibody has a strong ability to bind to a free Asp residue at position 1 of the β-amyloid; this characteristic prevents the recognition of an unprocessed amyloid precursor protein. Moreover, the 3D6 epitope is perceivable in all forms of Aβ, from soluble oligomeric species to compacted β-amyloid plaques. The supposed mechanism of action is represented by the induction of Fc-receptor-mediated microglial phagocytosis, with the result of the neutralization and removal of Aβ plaques [69,103].

A substantial body of the comprehensive preclinical research endorsing passive anti-Aβ immunotherapy for Alzheimer’s disease has relied on the use of 3D6. In mouse models of Aβ amyloidosis, a minor proportion of the peripherally administered antibody manages to enter the central nervous system. This antibody has demonstrated the ability to bind to amyloid plaques, reduce the plaque burden, ameliorate measures of synaptotoxicity, and enhance performance in mouse behavioral assays [102,104].

The phase I trial for Bapineuzumab aimed to identify the safety and tolerability of single ascending doses and describe the pharmacokinetic profile of this molecule to offer a preliminary evaluation for upcoming multiple-dose regimens in patients with mild to moderate Alzheimer’s disease. In this multicenter, randomized, third-party, unblinded, placebo-controlled, single-dosage study, male and female patients were divided into three active ascending dose groups (0.5 mg/kg, 1.5 mg/kg, and 5 mg/kg); bapineuzumab was administered intravenously. The MMSE test was used to evaluate whether there was a general cognitive improvement in each dose group compared to the placebo groups. A predetermined analysis of the MMSE change at week 16 was intended as the study’s exploratory efficacy endpoint, with the following results: both the 0.5 and 1.5 mg/kg dose groups had an increased MMSE score, while there was no effect for the 5 mg/kg dose group compared to the placebo group. As for adverse effects, 93% of patients reported at least one (mild to moderate in severity), but they were not attributed to the treatment. Three individuals receiving bapineuzumab at a dose of 5 mg/kg experienced MRI anomalies consistent with vasogenic edema, with one of them being symptomatic and associated with a small focus of microhemorrhage. This trial validated the safety of bapineuzumab at dosages of 0.5 and 1.5 mg/kg and offered an ideal dosage schedule every 13 weeks [101,104,105].

A phase II clinical study enrolled 234 individuals with mild to moderate Alzheimer’s Disease that received ascending dosages of bapineuzumab (0.15 mg/kg, 0.5 mg/kg, 1 mg/kg, 2 mg/kg) or placebo in a ratio of 8:7 for a period of 18 months. ADAS-Cog and Disability Assessment for Dementia (DAD) served as the instruments employed for the assessment of the efficacy of bapineuzumab, but the trial failed to identify any significant treatment differences. However, the APOE ε4 carrier status was possibly associated with differences in the treatment results. Thus, according to some clinical indicators, potential treatment differences in favor of bapineuzumab were observed among noncarriers [106]. Regarding safety, adverse effects were documented by 94% of the patients receiving bapineuzumab compared to 90% receiving a placebo; the most frequent adverse effects reported in >5% of the patients were vasogenic edema, back pain, anxiety, paranoia, vomiting, hypertension, weight loss, skin lacerations, muscle spasms, and gait disturbance. Only vasogenic edema was found to be dose-related. Also, 10 out of the 12 patients presenting vasogenic edema were individuals with the APOE ε4 allele, suggesting that future trials should test carriers at lower dose ranges [106,107].

Due to the previous phase II trial, which identified the possibility of different responses to treatment depending on the APOE ε4 carrier status, two phase III double-blind, randomized, placebo-controlled trials were initiated. The first one included 1121 patients who were carriers of the APOE ε4 (NCT00575055), and the second one involved 1331 noncarriers (NCT00574132). The primary outcome was to determine the effectiveness of the compound in comparison to a placebo from the initial evaluation to week 78 using ADAS-Cog11 and DAD as measurement scales of cognitive function. Moreover, the secondary key point was to identify the changes in three disease biomarkers: the whole brain volume, the brain amyloid burden by analyzing the results of PIB-PET (positron-emission tomographic amyloid imaging with the use of Pittsburgh compound B), and the cerebrospinal fluid phospho-tau concentrations. The carrier group received either 0.5 mg/kg intravenous bapineuzumab or placebo in a 3:2 ratio, while the noncarrier group received 0.5 mg/kg or 1 mg/kg or a placebo in a 3:3:4 ratio [108,109,110]. The safety characteristics of bapineuzumab were similar to those reported in previous phase 1 and 2 studies. Doses of 0.5 and 1 mg/kg were generally well-tolerated, but dose-dependent treatment-emergent adverse events (TEAEs) were observed. ARIA-E was considered a dose-dependent TEAE, as it appeared more often in the 1 mg/kg dose group than in the 0.5 mg/kg group. Moreover, ARIA-E appeared in 16.7% of APOE ε4 carriers who received 0.5 mg/kg bapineuzumab compared to 4.9% of noncarriers who received the same dose regimen [108]. In terms of the primary endpoint, there were no significant differences between the bapineuzumab groups (0.5 vs. 1 mg/kg group and carriers vs. noncarriers group) and the placebo groups. Regarding the results of the PIB-PET scans, taken at baseline, week 45, and week 71, in the carrier subgroup, there appeared to be a decrease in the rate of accumulation of Aβ, although not as significant as in the phase 2 trials. Moreover, reduced levels of CSF phospho-tau concentrations were observed in this group. As for the noncarrier group, no change in the amyloid burden on PIB-PET and no significant decrease in CSF phospho-tau concentrations were observed. Even though the biomarker results from the carrier study were positive at some levels, both trials failed to identify the benefit of bapineuzumab regarding clinical outcomes [108,109].

While the behavior of monoclonal antibodies typically adheres to the nonlinear process of TMDD, the clinical doses are frequently sufficient to saturate the nonlinear phase, resulting in apparent linearity in pharmacokinetics. This observation aligns with our experiences with other monoclonal antibodies. Evaluating this in the case of bapineuzumab could, to some extent, be inferred from its minimum effective serum concentrations. However, establishing these concentrations is challenging due to the unmet clinical endpoints in phase 3 trials. Given that bapineuzumab primarily binds to a soluble ligand, its PK remains unaffected by this binding, particularly because the binding site is in the brain. Additionally, prior phase 1–2 data of bapineuzumab, administered intravenously, displayed linear PK across a dose range of 0.15 to 5 mg/kg. It is noteworthy that no interactions were anticipated between bapineuzumab and APOE ε4 apolipoproteins and their receptors. Consequently, APOE ε4 carrier status is not expected to impact the PK of bapineuzumab, as confirmed by the results of the population PK analysis [110].

The analysis identified body weight as a significant factor affecting the systemic exposure to bapineuzumab. Although non-Caucasian subjects exhibited slightly higher clearance than Caucasian subjects, this difference was not considered clinically significant in terms of systemic exposure. Notably, we are unaware of any other monoclonal antibodies for which ethnicity has been identified as a PK covariate after accounting for body weight effects. Concomitant medications did not have relevant effects on bapineuzumab clearance, and the immune response as a covariate was not assessed due to the absence of anti-drug antibodies [110].

#### 2.1.6. Solanezumab

Solanezumab (LY2062430) is a humanized monoclonal IgG1 antibody that binds to the mid-domain epitope of the Aβ peptide (Aβ 16–26), which is found in oligomers and fibrils; therefore, it only targets soluble monomers and perhaps low-n oligomers.

The binding between solanezumab and the monomer prevents the formation of amyloid plaque in laboratory studies. Moreover, in preclinical studies, it was noted that solanezumab increases the clearance of Aβ from the brain through a phenomenon called the “peripheral sink”: high-affinity antibody-Aβ peptide complexes are formed and transferred to plasma. In transgenic mouse models, this mechanism reduces the Aβ burden and improves cognitive function [69,111,112].

It is worth mentioning that solanezumab’s Phase I trial in mild to moderate Alzheimer’s patients (0.5 mg/kg to 10 mg/kg doses) showed good tolerability, with minor infusion-related symptoms at the highest dose. Dose-dependent increases in plasma and CSF amyloid β were observed, but no cognitive improvement was noted. In Phase II, various dose regimens (100 mg every 4 weeks, 100 mg weekly, 400 mg every 4 weeks, 400 mg weekly) showed no adverse effects directly linked to solanezumab. Aβ levels increased dose-dependently, and the rise in unbound Aβ42 in CSF suggested solanezumab’s potential to mobilize Aβ42 from plaques, but no cognitive improvement was evident on the ADAS-Cog evaluation [113,114,115,116].

Two completed Phase III, randomized, double-blind clinical trials, EXPEDITION 1 (NCT00905372) and EXPEDITION 2 (NCT00904683), enrolled 2052 patients with mild to moderate Alzheimer’s disease to undergo monthly infusion treatments of 400 mg solanezumab or a placebo for a period of 18 months. Initially, the primary objectives of both trials were a change from the baseline in ADAS-Cog11 and ADCS-ADL (Alzheimer’s Disease Cooperative Study–Activities of Daily Living scale) by week 80. The FDA granted approval for the modification of the main goals of EXPEDITION 2 to a change from baseline to week 80 in ADAS-Cog 14 after data analysis from the EXPECTATION 1 trial showed a reduction in cognitive decline in mild forms of Alzheimer’s disease using scores on the 14-item cognitive subscale of the Alzheimer’s Disease Assessment Scale. Even so, both studies failed to demonstrate the significant efficacy of solanezumab in slowing cognitive deterioration, and the primary objectives of the trial were not achieved [116,117,118,119]. As for biological markers and neuroimaging outcomes, the tests performed at baseline included identifying the APOE ε4 carrier status, the plasma and CSF levels of Aβ and tau protein, the brain volumetric MRI, and the PET. After 80 weeks, they were retaken with the following results: no treatment effect on total brain or hippocampal volumes (MRI) or on amyloid accumulation (F-florbetapir-PET). No concrete difference in the efficacy of the treatment was identified between APOE ε4 carriers and noncarriers. The changes observed in Aβ levels (both bound and unbound) in plasma and CSF were expected considering the mechanism of action of solanezumab, which targets soluble brain amyloid. Regarding safety, the prevalence of severe adverse effects was low, and no association between any event and solanezumab treatment was identified. ARIA with edema or hemorrhage appeared in 0.9% and 4.9%, respectively [116] (Table 6).

Another double-blind, placebo-controlled, Phase III clinical trial, EXPEDITION 3 (NCT01900665), was initiated in 2013. It enrolled 2100 patients with mild dementia due to Alzheimer’s disease and proven amyloid burden from PET scans. After missing the primary endpoint (change from baseline in ADAS-Cog), the secondary measured outcomes were deemed descriptive and with no statistical significance, so the trial was terminated [120,121].

A pharmacokinetic model for solanezumab, based on data provided by the EXPEDITION trials, revealed typical population values for clearance, intercompartmental clearance, the central volume of distribution, and the peripheral volume of distribution. The estimated apparent binding coefficient between solanezumab and Aβ40 suggested a substantial reduction in free plasma Aβ at the steady state. The model indicated that Asian race, body weight, and gender had statistically significant but not clinically meaningful influences on solanezumab’s PK. The subsequent pharmacokinetic/pharmacodynamic (PK/PD) model predicted a reduction of approximately 91% in free plasma Aβ at the steady state. Notably, the study found no clinically meaningful patient-specific factors affecting solanezumab exposure, suggesting consistent exposures and drug effects across various patient characteristics in the studied AD population [122].

EXPEDITION PRO, a Phase III, multicenter, placebo-controlled trial, aimed to evaluate the efficacy and safety of solanezumab compared to a placebo for patients with prodromal AD. The study was supposed to include 2450 subjects diagnosed with either prodromal AD per the International Working Group (IWG) criteria or with mild cognitive impairment due to AD per the National Institute on Aging-Alzheimer’s Association (NIA-AA) criteria. After only 26 enrollments, the trial was terminated because there was inadequate scientific evidence to potentially show a tangible benefit of solanezumab [119,123,124,125].

The most recent Phase III, randomized clinical trial for solanezumab (NCT02008357) is a secondary prevention trial that targeted individuals with preclinical stages of Alzheimer’s disease. The criteria for inclusion were: an age between 65 and 85, evidence of amyloid plaque burden on 18F-florbetapir PET, a CDR score of 0, and an MMSE score of 25 or more. Unfortunately, the study failed to demonstrate a decrease in cognitive decline (measured using the Preclinical Alzheimer Cognitive Composite score) compared to the placebo, and the primary objective of the trial was not achieved [119,126].

#### 2.1.7. Gantenerumab

Gantenerumab (RO4909832) is a human IgG1 antibody specifically engineered to have an extremely strong affinity for a specific shape or conformation of Aβ fibrils [124]. This antibody binds to regions of both the N-terminal and central amino acids of Aβ (amino acids 3–11 and 18–27), which are key components of the amyloid plaques associated with Alzheimer’s disease. In contrast to bapineuzumab and solanezumab, which represent humanized adaptations of murine antibodies, gantenerumab stands out as the fully human anti-Aβ monoclonal antibody to progress into clinical development [124].

In preclinical studies, gantenerumab exhibited favorable pharmacodynamic characteristics, efficiently passing the blood–brain barrier with high affinity to cerebral amyloid plaques. Therefore, it is able to break them down by recruiting microglial cells and activating phagocytosis. Also, long-term treatment in vivo resulted in a reduction in the amyloid plaque load without the emergence of antidrug antibodies. In terms of pharmacokinetics, gantenerumab exhibited linear profiles, and its binding to Aβ plaques persisted for over two months in transgenic AD models. Importantly, gantenerumab shows a strong preference for interacting with aggregated Aβ found in both brain tissue and blood vessels [124,125]. It is important to note that gantenerumab does not have an impact on the levels of Aβ in the bloodstream [124,125]. Functional studies, including assessments of synaptic plasticity and behavioral outcomes, suggested the potential for mitigating cognitive deficits in AD models. While gantenerumab’s impact on the Morris water maze test results was inconclusive, it demonstrated an ability to attenuate the inhibition of the long-term potentiation caused by soluble Aβ42 aggregates. Notably, gantenerumab treatment did not induce neurological or motor impairments even after an extended duration of administration in preclinical studies [127]. 

In all four Phase 1 trials conducted internationally to assess the safety and tolerability of gantenerumab, the antibody was described as safe and well-tolerated. However, these trials raised concerns regarding ARIA. For instance, in one published study from 2012, it was reported that among six patients in the highest-dose group, two of them exhibited focal areas of inflammation or vasogenic edema when examined using MRI scans. These abnormalities were observed in brain areas where there was a significant reduction in amyloid levels. While the antibody showed promise in terms of safety and tolerability, the occurrence of ARIA highlights the importance of closely assessing its impact on patients undergoing treatment [67].

In February 2020, gantenerumab was announced to not have achieved the main goal in a phase II clinical trial conducted as part of the Dominantly Inherited Alzheimer Network Trials Unit (DIAN-TU) study (NCT04623242). This trial involved individuals with inherited Alzheimer’s disease [67]. Subsequently, another failed Phase II trial, DIAN-TU-001 (NCT01760005), was conducted specifically in individuals with mutations associated with early-onset AD [67,125]. In this trial, gantenerumab treatment resulted in several noteworthy findings. Gantenerumab treatment significantly decreased the presence of Aβ plaques in the brain and the treatment also led to reductions in cerebrospinal fluid biomarkers, including total tau and phospho-tau. Gantenerumab treatment attenuated the increases in the neurofilament light chain, suggesting a potential protective effect on neurons [125]. However, despite these positive biomarker outcomes, gantenerumab did not show significant benefits for cognitive measurements, and also, ARIA, particularly ARIA edema, was noted in 19.2% of the individuals [67,125,126].

#### 2.1.8. ACU193

ACU193 is a humanized monoclonal antibody belonging to the IgG2 subclass. It is an affinity-matured version derived from its murine parent, ACU3B34, an IgG1 antibody. Its property to selectively bind to soluble Aβ oligomers is presently undergoing evaluation in a multicenter phase 1 clinical trial involving individuals with mild cognitive impairment and mild Alzheimer’s disease [67,87,126,127].

ACU193 underwent preclinical investigations to assess its central pharmacologic activity in transgenic models of AD. Administered peripherally, ACU193 demonstrated significant behavioral improvements in both pre- and post-plaque accumulation scenarios, as observed in blinded studies. These studies revealed an enhanced performance in water maze trials, reduced hyperactivity in transgenic mice, and improved behavioral deficits even before detectable amyloid deposition in young mice. The findings suggest that ACU193 has the potential to intervene at early stages, preventing irreversible synaptic loss and associated behavioral impairments [127].

ACU193 is anticipated to carry a low risk of ARIA due to its minimal or absent binding to amyloid plaques, its apparent lack of interaction with the vascular amyloid, and its non-inducing effect on microhemorrhage in preclinical investigations. Given its favorable pharmacological characteristics, ACU193 is presently undergoing evaluation in a phase 1 clinical trial named INTERCEPT-AD. The decision to proceed or halt the trial will be based on considerations including safety and tolerance, pharmacokinetic assessments, and the demonstration of effective target interaction [87,88].

#### 2.1.9. Trontinemab

Trontinemab (RO7126209) is a 2 + 1 bispecific monoclonal antibody designed to bind bivalently to Aβ plaques and monovalently to human TfR1 (transferrin receptor 1). It is essentially an enhanced version of gantenerumab, designed to exhibit superior penetration of the blood–brain barrier. It exhibits similar affinity to fibrillar Aβ as well as Aβ plaques on human AD brain sections, suggesting that its gantenerumab paratope maintains binding affinity and selectivity to various Aβ aggregation species. The TfR1-binding cross-Fab moiety of trontinemab specifically binds to a preformed complex of the recombinant extracellular domain of human TfR1 and holo-transferrin in a monovalent mode without disrupting the endogenous ligand–TfR1 interaction. The bispecific monoclonal antibody shows comparable affinity to cynomolgus and human TfR1. Trontinemab, in lower doses than gantenerumab, effectively activates effector cells, as evidenced by induced cytokine secretion from pre-activated monocytes in the presence of Aβ [128]. 

The enhanced bioavailability of trontinemab has undergone evaluation in a phase 1 trial involving healthy individuals and is presently under investigation in another phase 1 study that involves patients with mild cognitive impairment and mild Alzheimer’s disease who have positive PET-amyloid scans [87,128].

#### 2.1.10. Remternetug

Remternetug (LY3372993) is an IgG1 humanized antibody, specifically designed to target the N3pGlu peptide of the amyloid beta A4 precursor protein (APP) Aβ42. It is specifically designed to target the pyroglutamate modification found on the third amino acid of the amyloid-beta peptide, a feature exclusive to amyloid plaques in the brain. Currently, it is undergoing evaluation in a phase 1 clinical trial involving individuals with mild cognitive impairment or mild Alzheimer’s disease. Preliminary findings indicate that a monthly intravenous dose of 2800 mg effectively converts participants from amyloid PET-positive to amyloid PET-negative within a span of 3 months. Additionally, remternetug is also the subject of a phase 3 study, TRAILRUNNER-ALZ1, which compares its efficacy to a placebo in early symptomatic AD over the course of one year, with both intravenous and subcutaneous administration options [87,129].

### 2.2. Tau Pathology

#### 2.2.1. Bepranemab

**Figure 3:**
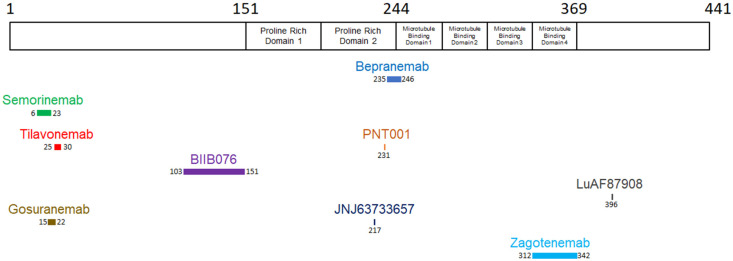
Tau domains targeted by monoclonal antibodies [67].

Bepranemab (UCB0107) is a monoclonal IgG4 antibody that has been humanized to target the central portion of tau, specifically amino acids 235 to 246 [67]. Antibodies targeting the midregion of tau appear to have greater potential for disrupting the transmission of pathogenic and aggregated tau between cells when compared to antibodies that aim at the N-terminal region of tau [67,130] (Table 7) (Figure 3).

Bepranemab, emerges as a promising avenue in preclinical investigations aimed at tackling tau pathology. The strategy of targeting a region near the microtubule-binding domain shows promise in effectively hindering the spread of pathogenic tau. The antibody exhibited enhanced efficacy in preventing pathological tau seeding and aggregation in a cell-based assay, surpassing the performance of alternative antibodies. Notably, in experiments with transgenic mice, it successfully averted the onset of tau pathology induced by injecting tau seeds sourced from Alzheimer’s disease brain extracts. Additionally, it demonstrated the capability to impede the dissemination of tau pathology to distant brain regions. Another significant aspect is its human IgG4 isotype, indicating a potential independence from microglial phagocytosis for the elimination of tau bound to the antibody. This distinctive characteristic introduces an additional layer of complexity to its potential therapeutic effectiveness in alleviating neurodegenerative processes associated with tau [131].

Commencing in February 2018, a Phase 1 study (NCT03464227) initiated the exploration of bepranemab’s PK and PKPD attributes. This study, focusing on healthy subjects, extended over a 20-week period, involving the administration of up to seven doses of bepranemab. The primary objective was the assessment of adverse effects, with secondary outcomes encompassing antibody exposure in both blood and CSF, pharmacokinetic parameters, and immunogenicity. The trial results revealed the absence of treatment-related adverse events and the non-detection of anti-drug antibodies. Notably, the study demonstrated a dose-dependent increase in bepranemab levels in both serum and CSF. Significantly, the CSF/serum ratio remained consistent across varying doses, providing crucial insights into the antibody’s distribution [131].

Currently, there is an ongoing phase 2 trial, called Together (clinical trial NCT04867616), that aims to assess the effectiveness, safety, and tolerance of bepranemab in individuals with mild Alzheimer’s disease or early-stage Alzheimer’s disease [67,132,133] (Table 8).

In the initial phase of the investigation, participants will be assigned randomly to one of three groups. These groups will receive either a placebo through intravenous infusion or one of two different doses of the experimental drug over a span of 20 months [134]. Subsequently, during the following phase, individuals already on the experimental drug will continue with their existing dosage, while those who were initially given the placebo will be randomly allocated to one of two different doses of the experimental drug for a duration of six months. All participants will undergo brain imaging using PET and MRI at the study’s commencement, as well as at the 14-month and 20-month marks. Additionally, they will complete various cognitive assessments and may choose to provide blood and CSF samples. Researchers will also monitor alterations in cognitive abilities and daily functioning, observe shifts in the levels of proteins associated with Alzheimer’s disease, and monitor the concentration of the experimental drug in the bloodstream. Furthermore, the safety of the drug will be assessed throughout the treatment period and for an additional year after the final dose. The anticipated completion date for this trial is November 2025 [67,132].

#### 2.2.2. Semorinemab

Semorinemab (RO705705) is a humanized IgG4 antibody targeting extracellular tau. It possesses the ability to bind to all six human tau isoforms (amino acids residues 6–23). In addition, it provides protection to neurons against the neurotoxicity caused by tau oligomers in co-culture settings involving neurons and microglia [67].

In preclinical studies, a murine version of semorinemab showcased promising outcomes by reducing tau-related toxicity in a cell culture and mitigating tau accumulation in a transgenic mouse model of tauopathy. However, the translation of these preclinical successes to the clinical setting has encountered challenges. A phase 1 study demonstrated dose-dependent target engagement and a favorable safety profile, prompting further clinical investigations in early Alzheimer’s disease (AD). Yet, the discrepancy in preclinical efficacy, particularly regarding the murine surrogate’s effectiveness in reducing tau accumulation, raises questions. Possible explanations include variations in tau species responsible for tau spread in prodromal to mild AD, patient-specific epitope targeting considerations, and diverse mechanisms of cell-to-cell tau spread in patients compared to preclinical models. The complexities underscore the need for a nuanced understanding of tau pathology and the challenges in translating preclinical findings to clinical outcomes [67,133,135].

#### 2.2.3. Tilavonemab

Tilavonemab (ABBV-8E12) is a monoclonal IgG4 antibody designed to target the aggregated, extracellular form of pathological tau by binding to the N-terminus of tau (amino acids residues 25–30).

Tilavonemab, distinguishes itself from other anti-tau antibodies through a mechanism of action that does not necessitate uptake into neurons. Preclinical investigations involving the murine version of this antibody revealed its ability to block seeding in a cell-based tau sensor assay and hinder the uptake of Alzheimer’s disease-derived tau by primary neurons. In the context of P301S tau-transgenic mice, the parental antibody demonstrated notable outcomes, including the reduction in brain neurofibrillary pathology, insoluble tau, microgliosis, and brain atrophy. These findings underscore the distinctive attributes of the murine version of tilavonemab in preclinical models, emphasizing its potential as a promising anti-tau therapeutic candidate with a unique mechanism of action [130].

This medication successfully underwent safety validation in a phase I trial (NCT02880956) [67,136,137,138]. However, the subsequent phase II trial, which aimed to evaluate the effectiveness and safety of tilavonemab in 453 patients with early Alzheimer’s disease, did not yield the anticipated results. Consequently, tilavonemab has been discontinued as a treatment for Alzheimer’s disease (NCT02880956) [137,138,139].

#### 2.2.4. BIIB076

BIIB076 (NI-105) is a human IgG1 antibody that specifically targets the mid-domain of tau (amino acids 103–151).

In preclinical investigations, BIIB076 exhibits high-affinity binding to recombinant tau from both human and monkey sources, demonstrating subnanomolar affinity. Administered in young species monkeys, BIIB076 exhibits a half-life ranging from 8 to 11 days in blood. While the concentration in CSF reaches its peak within 24–48 h post-administration, it is noteworthy that CSF levels are approximately 1000 times lower than those in plasma. Notably, BIIB076 leads to an elevation in the total tau protein in plasma, but no significant impact on total tau in CSF is observed. Unbound tau in CSF experiences a notable reduction 24 h after antibody administration. Conducting a toxicity study in order to assess the safety profile, it was noted that the highest doses employed, both total and free tau in CSF, exhibited reductions. Encouragingly, the study reported no instances of toxicity or pathology associated with BIIB076, reinforcing its favorable safety profile [131]. The antibody successfully completed a phase I clinical trial (NCT03056729) [67,138,139,140,141]. BIIB076 demonstrated engagement with its target, leading to a 50% reduction in mid-region-bearing tau levels in the cerebrospinal fluid within one week after administration. This reduction remained significant for up to three weeks. The majority of adverse incidents documented throughout the trial were of mild to moderate intensity and were more frequent at higher doses. Common side effects included headaches, dizziness, nausea, vomiting, and lowered blood pressure. However, in July 2022, the discontinuation of the clinical trial for this molecule was announced [139,140,141,142].

#### 2.2.5. Lu AF87908

Lu AF87908 is a humanized IgG1 antibody developed to target phosphorylated tau at the serine 396 site.

In the context of preclinical investigations, the parental murine of this particular antibody exhibited a distinct affinity for binding to hyperphosphorylated tau aggregates within the brain. It effectively curtailed the ability of tau derived from the brain to aggregate in both cultured neurons and transgenic mice. The antibody played a facilitating role in the internalization and subsequent lysosomal degradation of pathological tau aggregates derived from mouse brains. In its humanized configuration, the antibody selectively targeted phosphorylated tau in postmortem brains afflicted by Alzheimer’s disease and primary tauopathies. Additionally, it demonstrated a preventive effect on the initiation of aggregation induced by brain extracts [131,132,133].

At present, research on this antibody remains in the preliminary phases, and an ongoing phase I clinical trial is actively enrolling participants to assess the safety of a single dose of Lu AF87908 in both healthy individuals and individuals with Alzheimer’s disease (NCT04149860) [67,140,141,142,143,144].

#### 2.2.6. Gosuranemab

Gosuranemab (BIIB092) is a humanized IgG4 monoclonal antibody directed against the extracellular N-terminal region of tau (residues 15–22) [142,143].

In the field of preclinical research, it was discovered that the addition of external eTau elevated the production of Aβ and triggered heightened neuronal activity in primary human cortical neurons. An interesting finding emerged when an antibody was utilized to counteract the effects of eTau, leading to a decrease in Aβ production. This pattern was consistently observed in two separate models involving human tau transgenic mice. The antibody exhibited strong binding capabilities to diverse tau forms, encompassing monomers, fibrils, and insoluble tau [144,145,146,147].

It is worth mentioning that gosuranemab was considered to have potential therapeutic benefits for other tauopathies besides AD, such as progressive supranuclear palsy (PSP), corticobasal degeneration, argyrophilic grain disease, Pick disease, and Huntington’s disease. That is why the molecule was the subject of other clinical trials apart from those oriented towards AD. In 2015, a Phase 1b clinical trial evaluated the safety and tolerability of BIIB092 in patients with PSP. The results were favorable, with BIIB092 being considered well-tolerated in participants suffering from progressive supranuclear palsy. This trial was the basis for the Phase 2 PASSPORT (NCT03068468) trial, which started in 2017 [145,146,147,148,149]. However, in December 2019, due to a lack of efficacy, the development of the molecule for PSP, and other primary tauopathies, it was brought to an end (the AD research was continued) [145,146,147,148,149,150].

In May 2018, a phase 2 study called TANGO (NCT03352557) was launched, which involved more than 600 participants diagnosed with mild cognitive impairment resulting from either Alzheimer’s disease or mild Alzheimer’s disease. The study was designed as a randomized, double-blind, placebo-controlled trial and specifically recruited individuals who had a positive PET scan for amyloid. The primary objective of the placebo-controlled period of the trial was to determine the safety and tolerability of BIIB092 in the participants mentioned above. Other objectives included changes in the CDR-SB after the first one and a half years and to identify the possible development of anti-BIIP092 antibodies. The study was divided into two periods: the placebo-controlled phase, which ended in 2021, and the long-term extension phase, which was expected to last until 2024. Regarding the latter, the evaluation of the long-term safety of the molecule was the main goal. Patients were divided into four groups, each of them receiving either a low, medium, or high dose of the drug or a placebo; the medication was administered intravenously monthly, and the total duration of the treatment period was 76 weeks. After this period, patients entered the long-term extension period, and they all received BIIP092 [145,148,149,150,151,152]. After 1.5 years of the trial, it was announced that the molecule failed to achieve its primary endpoint of improving the CDR-SB results. Moreover, according to the negative results of ADAS-Cog13 (which was a secondary endpoint of the study), BIIP092 treatment led to cognitive decline in subjects who received the active molecule compared to the placebo. However, statistical significance was reached only by the patients who received the highest dose of gosuranemab. Due to the lack of efficacy, the study was discontinued, and the development of gosuranemab was terminated [145,149,150,151,152].

#### 2.2.7. Zagotenemab

Zagotenemab (LY3303560) is a humanized IgG4 antibody targeting the 7–9 and 312–342 amino acids of tau, derived from MCI-1. MCI-1 is an anti-Tau antibody that can bind to the premature pathological conformation of tau, which is soluble and formed before the building of paired helical filaments (PHF). Because the MCI-1 epitope tends to appear in neurons before the assembly of PHF but is not present in the normal brain structure, studies suggest that the MCI epitope’s appearance is one of the initial pathological alterations of tau in Alzheimer’s disease [136,153,154,155]. In preclinical investigations, the efficacy of the zagotenemab parental antibody has been highlighted through studies involving transgenic mice and non-human primates. When transgenic mice were treated with MC1, a notable reduction in phosphorylated tau levels and neurofibrillary pathology was observed. This effect was attributed to the clearance of tau/antibody complexes, facilitated either by microglia-dependent or neuronal-dependent mechanisms. Furthermore, it demonstrated a selective and high-affinity binding to tau aggregates as opposed to monomers. In non-human primates, the intravenous administration of LY3303560 exhibited a half-life of 13 days in serum, with a clearance rate of 0.15 mL/h/kg. Additionally, in a rat model, the concentration of LY3303560 in CSF was found to be the ten part of the plasma levels at just 1 day post-injection [131,133,154].

A phase 2 clinical trial (NCT03518073) started in 2018; it included 360 patients with early symptomatic AD (progressive deterioration of memory for at least six months). The patients received two different doses of LY3303560 (1400 mg or 5600 mg) or a placebo once every 4 weeks for 100 weeks. A change from baseline on the iADRS was the primary measured outcome. Secondary key points included a change from baseline on the ADAS-Cog13 Score, Alzheimer’s Disease Cooperative Study-Instrumental Activities of Daily Living Scale (ADCS-iADL) Score, CDR-SB Score, MMSE Score, brain aggregated tau deposition measured by PET scan, brain volume measured by MRI, and Columbia Suicide Severity Rating Scale (C-SSRS); also, identifying the number of patients that developed treatment-emergent anti-drug antibodies (TE-ADA) was a secondary measured outcome. The trial ended in August 2021 and was discontinued in October 2021 because the primary endpoint of the study was not achieved. As a result, the company decided to terminate the development of zagotenemab [154,156].

#### 2.2.8. PNT001

PNT001 is a humanized monoclonal IgG4 antibody that targets cis-pT231 tau (a cis-isomer of tau phosphorylated at threonine231). Cis-pThr231-tau has been found in brain tissue form individuals with Alzheimer’s disease (it is even considered one of the earliest detectable pathogenic conformations that appears) and chronic traumatic encephalopaty. Moreover, it was demonstrated that the cerebrospinal fluid levels of Cis-pThr231 are directly proportional with the severity of the brain injury. In mouse models of vascular dementia, the cis monoclonal antibody had beneficial effects on cognitive impairment and neurodegeneration [157,158,159,160].

In 2020, a phase I trial assessed the safety and tolerability of intravenously administrated PNT001 in healthy volunteers at dose levels that may have a potential therapeutic effect. Thereby, a phase Ib/II trial, this time in patients with Alzheimer’s disease, is expected in order to verify the efficacy of the treatment [160].

#### 2.2.9. RG7345

RG7345 is a humanized monoclonal antibody that is targeting the tau phosphoepitope serine422 (S422). When the phosphorylation appears at this spot, tau relocates towards the somato-dendritic part of the neuron instead of microtubules, which is considered pathological [161]. 

In 2015, a phase 1, randomized, placebo-controlled, single ascending dose study was initiated in order to evaluate the safety, tolerability, and pharmacokinetics of RG7345 in 48 healthy male participants. However, in October 2015, the study was discontinued without further information [161,162].

#### 2.2.10. JNJ-63733657

This monoclonal humanized IgG1 antibody, JNJ-63733657, is designed to identify the microtubule binding region of the tau protein (Thr217). In preclinical investigations, its impact on cell cultures was demonstrated, showcasing its ability to eliminate pathogenic tau. Moreover, in murine models, it exhibited inhibitory effects on the dissemination of tau [163,164,165]. 

Between 2017 and 2020, a phase 1 clinical trial was conducted in Europe. The primary objective of this trial was to assess the safety and tolerance of JNJ-63733657, and it involved the participation of 72 individuals. In the initial phase of this trial, a single, increasing dose of the experimental drug was administered via intravenous infusion to healthy volunteers. Subsequently, in part two of the trial, multiple ascending doses of the drug were infused into participants diagnosed with prodromal or mild Alzheimer’s disease. The study’s outcomes encompass a range of factors, including the occurrence of adverse events, the concentration of JNJ-63733657 in both blood and CSF, pharmacokinetic parameters related to the antibody’s accumulation, distribution, and clearance in the body, and the detection of host antibodies against JNJ-63733657 [166,167,168,169,170]. The results of the single-dose study indicated that there were no immediate safety concerns associated with the treatment [168]. The serum pharmacokinetics exhibited a linear relationship with the administered dose, and only a small fraction, specifically 0.2%, was detected in the CSF [166,167,168,169,170].

In November 2020, the results of the multiple dosing study were presented [169]. The pharmacokinetics of the drug were found to be consistent whether administered to healthy volunteers or to those with Alzheimer’s disease who received three monthly doses [167,168,169]. The drug was generally well tolerated, with the most common complaints being back pain and headaches. Moreover, whether given as a single dose or multiple doses, there were dose-dependent reductions observed in free p217 tau levels in the CSF [166,167,168,169,170].

In January 2021, a phase 2 clinical trial was initiated, involving individuals who exhibited early symptoms of AD and had received a positive tau PET scan. To be eligible for enrollment, patients needed to have a CDR score of 0.5 and report experiencing subjective cognitive decline within the past six months. These participants were assigned to receive either a low or high dose of the experimental antibody or a placebo that matched their respective treatment every four weeks, with the treatment period extending up to 4.5 years. Initially, the primary focus of the study was on assessing cognition, using the ADAS-Cog 13 scale as the main metric for this assessment. Secondary outcome measures included various standard assessments of cognition and functional capabilities, as well as evaluating the extent of the tauopathy burden through PET scans and CSF tau levels. Safety and pharmacokinetics were also closely monitored. Later in 2021, there was a change in the primary endpoint of the study to the integrated Alzheimer Disease Rating Scale, which combines measures of both cognition and function. This clinical trial is anticipated to continue until 2025 [171].

#### 2.2.11. E2814

This humanized monoclonal IgG1 antibody is designed to target the HVPGG epitope within the microtubule-binding domain, situated near the midpoint of the tau protein (residues 299–303, 362–366). This region plays a significant role in the formation of tau tangles and is closely associated with the initiation and dissemination of pathological tau aggregates [172]. The primary objective of this antibody is to bind to extracellular tau, thereby obstructing the transmission of harmful tau species between cells and facilitating their removal through microglial clearance mechanisms. In a model of transgenic mice, this molecule was found to lessen the deposition of tau aggregates in mice injected with tau fibrils. In non-human primates, it displayed a binding pattern dependent on the dosage to mid-domain tau fragments, leading to a reduction in the levels of free tau containing the mid-domain [172,173,174].

In November 2021, there was an amendment to the design of the DIAN-TU study, allowing for the inclusion of the anti-amyloid antibody lecanemab [175]. According to the trial registry, the study is set to enroll 168 participants who have varying cognitive statuses, including normal cognition, mild cognitive impairment, or mild dementia. Individuals with mild cognitive impairment or dementia will initially receive open-label intravenous lecanemab for a period of 24 weeks [175,176]. Following this, they will be randomized into two groups. One will receive intravenous E2814 or placebo in addition to lecanemab for the remaining four years of the trial [175,176,177]. Participants with normal cognition, on the other hand, will begin with E2814 or a placebo for the first year, after which they will add open-label lecanemab to their treatment regimen. The primary endpoint of the trial, within the symptomatic cohort, is the assessment of tau spread measured using tau PET scans [175]. The other outcomes studied were changes in a cognitive composite, amyloid PET results, and CSF neurofilament light chain levels. In the asymptomatic population, the main focus is the modification of the CSF ptau217/total tau ratio [126,175]. The study is anticipated to continue until 2027. The primary goal is to assess the potential benefits of anti-tau therapy when administered alongside anti-amyloid treatment [126,175,176,177,178,179,180,181].

## 3. Discussion

The rise of passive immunotherapy in Alzheimer’s disease treatment has been thoroughly discussed in light of recent trials and their outcomes. The collective feedback from these recent trials presents a mixed perspective on the potential of passive immunotherapy for AD. However, it is essential to note that the failure of a specific trial should not be taken as a reason to abandon the pursuit of passive immunization strategies. Instead, we should view these trials as valuable sources of learning [182].

We would like to emphasize the pivotal role of epitope specificity in determining the efficacy of antibodies targeting Aβ. Notably, a study on transgenic mice revealed that antibodies directed against the marginal 11 epitopes situated at the N-terminal of Aβ demonstrate significant efficacy in inducing plaque clearance, both in terms of plaque reactivity and ex vivo efficacy. This observation is further corroborated by in vivo immunization experiments, where antibodies targeting N-terminal epitopes 1–5, 3–9, and 5–11 substantially reduce the amyloid burden. In contrast, antibodies against a C-terminal epitope (15–24) prove to be ineffective in mitigating the amyloid burden or neuritic pathology. The study challenges conventional assumptions by revealing that capturing soluble Aβ is not necessary for reducing neuritic pathology, as exemplified by the unique efficacy of antibodies against Aβ3–9, which exhibit robust plaque reactivity but a weak recognition of soluble peptide. These findings provide valuable insights that can guide the development of more targeted and effective therapeutic interventions for Aβ-related conditions, emphasizing the critical need for epitope-specific considerations in antibody design and evaluation [183].

The same study also delves into the crucial interplay between the IgG subclass and efficacy in the context of the antibody-mediated clearance of Aβ plaques associated with Alzheimer’s disease. The research underscores the significance of Fc receptors in the phagocytotic activity of microglia, revealing that IgG2 antibodies, with a higher affinity for Fcγ receptors, exhibit superior efficacy in reducing plaque burden compared to IgG1. By leveraging six monoclonal antibodies of different IgG isotypes, all directed against the same Aβ epitope, the study demonstrates that not only the affinity of antibodies for Aβ but also their ability to trigger Fc-mediated plaque clearance is crucial for optimal therapeutic outcomes. Intriguingly, while IgG2 antibodies efficiently reduced plaque levels, IgG1 antibodies exhibited a lower efficacy in an ex vivo assay with primary mouse microglial cells. In a more extended in vivo study, IgG2 antibodies displayed a trend toward a greater reduction in total Aβ levels and significantly outperformed IgG1 subclass antibodies in protecting against neuritic dystrophy [183].

When it comes to anti-tau antibodies, targeting of the N-terminus in clinical trials has prompted concerns regarding their efficacy, primarily stemming from the observed lower levels of extracellular tau in comparison to intracellular tau. Mass spectroscopy studies have provided evidence that the majority of tau in the CSF lacks both N and C-termini, imposing an additional limitation on the suitability of N-terminal antibodies by diminishing the available target pool. This limitation raises questions about the practicality of exclusively focusing on the N-terminus, particularly given the challenges associated with targeting a tau region that may be less accessible or abundant in the extracellular environment. In contrast, antibodies designed to function extracellularly and directed towards the mid-region of tau appear to present a more promising avenue. By concentrating on this region, these antibodies may demonstrate greater efficacy in impeding tau seeding and propagation, offering a potentially more effective strategy for combating pathological tau species in neurodegenerative diseases [130,131,132,133].

The careful selection of IgG subclasses is a pivotal aspect of designing effective anti-tau antibodies, particularly within the intricate environment of the central nervous system, where microglia and neurons play crucial roles in tau clearance. Notably, human IgG1 stands out as the most effective isotype, demonstrating a superior ability to promote microglia phagocytosis and, consequently, facilitating the clearance of extracellular tau and antibody complexes. In contrast, IgG4 exhibits a comparatively lower efficacy in these processes. The ongoing clinical trials involving both IgG1 and IgG4 tau antibodies highlight the importance of carefully considering the unique characteristics of each isotype in the development of tau-targeted immunotherapies. This nuanced understanding of IgG subclasses is instrumental not only in optimizing efficacy but also in ensuring the safety and success of anti-tau antibody treatments [130,131,132,133].

By incorporating these lessons, we can gain a better grasp of the mechanisms of action and even the ideal dosages and optimal timing for administering treatment. Two more critical lessons to be taken into consideration for passive immunotherapy are the timing of treatment and the appropriate dosage. The stage of the disease at which therapy is administered plays a pivotal role in determining its effectiveness. This factor may elucidate some of the disappointing outcomes observed in the aforementioned trials. The rationale behind this notion makes sense from a biochemical standpoint. For instance, solanezumab is likely to have limited effectiveness in modifying the disease state once oligomeric Aβ and Aβ plaques have already formed, as these are not its targets. Additionally, determining the correct dosage for these agents is a critical challenge. Often, the administration of agents has been limited due to side effects like vasogenic edema, as seen with bapineuzumab. While it is crucial to minimize vasogenic edema, the real issue may have been related to dosing rather than a lack of efficacy. This suggests the potential for refining and developing these agents to increase the dosage for a more significant clinical impact while still minimizing side effects.

Moreover, it is worth mentioning that monoclonal antibodies employed in the treatment of Alzheimer’s disease exhibit intricate interactions with a spectrum of cellular proteins beyond the well-studied tau protein and beta-amyloid. These antibodies are designed to target various components involved in the complex cascade of events leading to neurodegeneration. For instance, they may interact with inflammatory mediators, such as microglial activation markers, to modulate the neuroinflammatory response associated with Alzheimer’s. Additionally, monoclonal antibodies might engage with synaptic proteins, promoting synaptic integrity and function. By influencing these diverse cellular targets, the antibodies aim to create a multifaceted impact on the disease process. The evolving understanding of these intricate interactions opens avenues for exploring novel therapeutic targets and refining treatment strategies to address the intricate network of factors contributing to Alzheimer’s disease [183]. Passive immunization is currently one of the most active areas of AD research, and it holds promise as a potential disease-modifying therapy for AD [182].

## 4. Conclusions

In conclusion, the use of monoclonal antibodies in the treatment of Alzheimer’s disease represents a pivotal step forward in the quest to combat this devastating neurodegenerative condition. The development of these passive immunotherapies for Alzheimer’s disease exemplifies the progress made in the field of neurodegenerative disease research, offering a targeted and specific approach to tackling the underlying pathological mechanisms. The body of research strongly suggests that monoclonal antibodies hold great potential in ameliorating the cognitive decline associated with AD by specifically targeting amyloid-beta and tau pathology. While safety concerns and individual variability must be thoughtfully considered, ongoing research and the pursuit of optimized treatment regimens offer avenues for mitigating these challenges. As our understanding of the disease continues to deepen, and as more monoclonal antibody treatments move through clinical trials and into clinical practice, we anticipate a paradigm shift in how we manage and potentially modify the course of Alzheimer’s disease. While challenges persist on the path to effective treatment, the promise of monoclonal antibodies as a vital weapon in the fight against AD is undeniable. With continued dedication to research, development, and patient care, we hope to see these innovative therapies enhance the quality of life for individuals affected by Alzheimer’s disease and move us closer to the realization of disease-modifying treatments that are urgently needed.

## Figures and Tables

**Table 1:** Comparative analysis of specific antibody epitopes [67].

Antibody	Antibody Species	Epitope Location	Binding Selectivity		
			Monomers	Oligomers	Fibrils/Plaques
Aducanumab	Human IgG1	3–7	-	+	+
Lecanemab	Humanized IgG1	1–16	-	++	+
Donanemab	Humanized IgG1	p3-7	-	-	+
Crenezumab	Humanized IgG4	13–24	-	+	+
Bapinezumab	Humanized IgG1	1–5	+	+	+
Solanezumab	Humanized IgG1	16–26	++	+	-
Gantenerumab	Human IgG1	2–11	-	+	-

-: no binding selectivity, +: moderate binding selectivity, ++: high binding selectivity.

**Table 2:** Antibodies for different stages of AD in ongoing studies [78].

Antibody	AD Stage
Aducanumab	Prodromal AD, mild AD
Lecanemab	Preclinical AD, prodromal AD, mild AD
Donanemab	Preclinical AD, prodromal AD, mild AD
Crenezumab	Prodromal AD, mild AD
Bapineuzumab	Mild AD, moderate AD
Solanezumab	Preclinical AD, prodromal AD, mild AD, moderate AD
Gantenerumab	Preclinical AD, prodromal AD, mild AD

**Table 3:** Immune response and pathological changes after immunotherapy [83].

Antibody	Pathological Changes
Aducanumab 10 mg/kg weekly	After 54 weeks, PET scans revealed a reduction in amyloid plaques.
Lecanemab 10 mg/kg Biweekly	PET scans detected a decrease in brain Aβ after 18 weeks The examination of cerebrospinal fluid identified an elevation in Aβ42 levels and a decrease in p-tau levels.
Bapineuzumab 0.5 mg/kg, 1.5 mg/kg, 5mg/kg weekly	No notable changes
Solanezumab 400 mg monthly	The analysis of cerebrospinal fluid revealed heightened concentrations of both total Aβ42 and Aβ40. Additionally, there was an increased level of free Aβ42, accompanied by a reduced level of free Aβ40.
Gantenerumab 1200 mg/4 weeks	After a period of two years, PET scans revealed a reduction in amyloid plaques.

**Table 4:** Cognitive evaluation after immunotherapy in AD [83].

Antibody	Clinical Changes
Aducanumab 10 mg/kg weekly	Slowed cognitive decline, measured by MMSE, CDR-SB No changes on NTB or FCSRT
Lecanemab 10 mg/kg Biweekly	Clinical symptoms displayed improvement, as indicated by measures such as ADCOMS, ADASCog14, and CDR-SB More favorable outcomes were observed in individuals with the APOE4+ genotype
Bapineuzumab 0.5 mg/kg, 1.5 mg/kg weekly	There were no significant improvements observed in measures such as CDR-SB, ADAS-Cog13, MMSE, FAQ, and FCSRT
Solanezumab 400 mg monthly	There were no significant improvements observed in measures such as CDR-SB, ADAS-Cog14, MMSE, and FAQ
Gantenerumab 1200 mg/4 weeks	No noteworthy improvements were detected based on assessments using CDR-SB, ADAS-Cog14, MMSE, and FAQ

MMSE = Mini-Mental State Examination, CDR-SB = Clinical Dementia Rating—Sum of Boxes, NTB = Neuropsychological Test Battery, FCSRT = The Free and Cued Selective Reminding Test, ADCOMS = Alzheimer’s Disease Composite Score, ADASCog = The Alzheimer’s Disease Assessment Scale-Cognitive Subscale, FAQ = Functional Activities Questionnaire.

**Table 5:** Half-time of the main anti-Aβ antibodies [83].

Antibody	Half-Life Time
Aducanumab	~24.8 days
Lecanemab	~9.5 days
Donanemab	~11.8 days
Crenezumab	~20 days
Bapinezumab	~21–26 days
Solanezumab	~20 days
Gantenerumab	~15 days

**Table 6:** Side effects of immunotherapies in AD [79,83].

Antibody	Clinical Changes
Aducanumab 10 mg/kg weely	High risk of ARIA-E and siderosis Higher risk was observed in individuals with the APOE4+ genotype
Lecanemab 10 mg/kg biweekly	Lower risk of ARIA-E and ARIA-H Higher risk of ARIA-E and ARIA-H was observed in individuals with the APOE4+ genotype
Bapineuzumab 5 mg/kg weekly	ARIA-E and micro-hemorrhage
Solanezumab 400 mg monthly	Very low risk of ARIA-E
Gantenerumab 1200 mg/4 weeks	High risk of of ARIA-E and ARIA-H

**Table 7:** Tau domains targeted by monoclonal antibodies [67].

Antibody	Antibody Type	Epitope Location
Bepranemab	Humanized IgG4	235–246
Semorinemab	Humanized IgG4	6–23
Tilavonemab	Humanized IgG4	25–30
BIIB076	Human IgG1	103–151
Lu AF87908	Humanized IgG1	396
Gosuranemab	Humanized IgG4	15–22
Zagotenemab	Humanized IgG4	312–342
PNT001	Humanized IgG4	231
JNJ-63733657	Humanized IgG1	217
E2814	Humanized IgG1	299–303, 362–366

**Table 8:** Immunotherapies for different stages of AD in ongoing studies [67,133].

Antibody	Stage of AD
Bepranemab	Prodromal AD, mild AD
Semorinemab	Mild AD, moderate AD
Tilavonemab	Prodromal AD, mild AD
BIIB076	Preclinical AD, prodromal AD, mild AD
Gosuranemab	Mild AD
Zagotenemab	Prodromal AD, mild AD
JNJ-63733657	Prodromal AD, mild AD
E2814	Mild AD, moderate AD

## Data Availability

The raw data supporting the conclusions of this article will be made available by the authors without undue reservation.
